# Treatment of Neonatal Hypoxic-Ischemic Encephalopathy with Erythropoietin Alone, and Erythropoietin Combined with Hypothermia: History, Current Status, and Future Research

**DOI:** 10.3390/ijms21041487

**Published:** 2020-02-21

**Authors:** Dorothy E. Oorschot, Rachel J. Sizemore, Ashraf R. Amer

**Affiliations:** Department of Anatomy, School of Biomedical Sciences, and the Brain Health Research Centre, University of Otago, Dunedin 9054, New Zealand; rachel.sizemore@otago.ac.nz (R.J.S.); ashraf.amer@otago.ac.nz (A.R.A.)

**Keywords:** erythropoietin, moderate hypothermia, perinatal hypoxic-ischemic encephalopathy, neonatal hypoxia-ischemia, anemia of prematurity

## Abstract

Perinatal hypoxic-ischemic encephalopathy (HIE) remains a major cause of morbidity and mortality. Moderate hypothermia (33.5 °C) is currently the sole established standard treatment. However, there are a large number of infants for whom this therapy is ineffective. This inspired global research to find neuroprotectants to potentiate the effect of moderate hypothermia. Here we examine erythropoietin (EPO) as a prominent candidate. Neonatal animal studies show that immediate, as well as delayed, treatment with EPO post-injury, can be neuroprotective and/or neurorestorative. The observed improvements of EPO therapy were generally not to the level of control uninjured animals, however. This suggested that combining EPO treatment with an adjunct therapeutic strategy should be researched. Treatment with EPO plus hypothermia led to less cerebral palsy in a non-human primate model of perinatal asphyxia, leading to clinical trials. A recent Phase II clinical trial on neonatal infants with HIE reported better 12-month motor outcomes for treatment with EPO plus hypothermia compared to hypothermia alone. Hence, the effectiveness of combined treatment with moderate hypothermia and EPO for neonatal HIE currently looks promising. The outcomes of two current clinical trials on neurological outcomes at 18–24 months-of-age, and at older ages, are now required. Further research on the optimal dose, onset, and duration of treatment with EPO, and critical consideration of the effect of injury severity and of gender, are also required.

## 1. Introduction

How to prevent brain damage due to hypoxic-ischemic encephalopathy (HIE) remains a question that needs an answer due to its serious sequels of neonatal death or severe intellectual, cognitive, and motor disabilities [1]. Perinatal HIE causes 23% of neonatal deaths [2,3] and affects 1.5–2 per 1000 births in developed countries, but the number affected increases to 26 per 1000 in resource-limited settings [4]. HIE involves a combination of decreased delivery of oxygen in the blood supply (i.e., hypoxia) and decreased blood flow (i.e., ischemia) to the brain.

Neonatal HIE can be subdivided into mild, moderate, and severe using modified Sarnat staging [5]. The Sarnat scale was introduced in 1976 [6]. In 1997, Thompson et al. developed a scoring system that was based on Sarnat and Sarnat (1976) but was simpler [7]. Nine symptoms were scored, including mental state, cranial nerve function (e.g., the ability to suck), motor ability and seizure activity. The outcome of treatment can depend on whether an infant experienced mild, moderate, or severe HIE.

The goals of management of neonates affected by HIE are:

1. Early identification, within 2–6 h of birth, of those at high risk. A high risk of HIE is likely in infants with fetal bradycardia (<100 beats/minute), an Apgar score of five or less at 5 minutes [8], a cord blood pH of 7 or less, and/or a base deficit of 16 or more [9]. The Apgar score enables a quick and accurate assessment of the respiratory, cardio-circulatory, and neurological condition of the newborn. The Apgar score at 10 minutes correlates with poor outcomes following HIE [10].
2. Adequate perfusion of the brain through supportive care. The supportive care can involve the provision of oxygen, volume expanders, ionotropes, diurectics, and antibiotics (see also Section 5).
3. Amelioration of the process of ongoing brain injury through neuroprotective and neurorestorative interventions [9,11]. Neuroprotective interventions are delivered within 6 h of HIE, while neurorestorative interventions have a delayed onset.

The current standard of care for moderate to severe HIE at term in resource rich settings is to apply moderate hypothermia (HT; a temperature decrease of 2–5 °C) that maintains a core body temperature of 33.5 °C for 72 h, commenced within the first 6 h of life [12,13,14]. Extending the period of HT and/or applying deeper cooling has not yielded any additional benefit [15].

Especially in severe HIE, HT alone is not enough to reduce the mortality nor avoid major neurodevelopmental disabilities [14,16]. A meta-analysis showed that 40% of infants with HIE who were treated with HT either died or suffered moderate to severe disabilities [17]. The authors of a recent review in 2017 concluded that HT has been the most important recent innovation in the treatment of HIE at term, although there is still a large number of infants for whom this therapy is ineffective [1].

Consequently, combining other neuroactive agents with HT to protect or restore brain damage secondary to HIE is vital and is the focus of current research for HIE. Currently, two of the most attractive adjunct therapies are xenon or erythropoietin (EPO) [18,19]. This is due to their biological properties and their safety in sick infants [18,19]. We recently reviewed the current literature on combining HT with xenon. We concluded that, while preclinical research provides supportive evidence, the routine use of this combined therapy awaits more clinical trials and the overcoming of major logistical constraints in the clinic to achieve timely post-hypoxic delivery of xenon [11]. Here we review the use of HT in combination with EPO for the treatment of perinatal HIE at term. Timely post-hypoxic delivery is not a major logistical constraint for EPO.

There is evidence that some EPO-treated preterm infants have improved neurodevelopmental outcomes. Hence, this literature is also reviewed and compared with term infants. This literature provides a useful guide regarding effective doses of EPO and the effect of infant age (see Section 7).

The use of HT in preterm infants, has caveats, however. These include hyperglycemia and a higher mortality [20]. Thus, the main focus of this review is the use of EPO alone, and of EPO in combination with HT, for the treatment of perinatal HIE at term.

This review builds on the work of McPherson and Juul (2010) [21] by describing new results that have been gained over the past 10 years from animal studies and clinical trials. We also include a novel section on the history of EPO over the past century.

## 2. EPO: Brief History and Synopsis of Functions

EPO is a glycoprotein regulating red blood cell production (i.e., erythropoiesis) [22]. More generally, EPO provides a mechanism to maintain or re-establish the function of all other cells in challenging physiological conditions (e.g., hypoxia) [23]. The importance of EPO is evident from the awarding, in 2019, of the ‘Nobel Prize in Physiology or Medicine’ to the three physician scientists, Kaelin Jr, Ratcliffe and Semenza, who discovered the three key molecular elements of EPO’s hypoxia signalling process [24]. Further details of this process are provided below.

**Figure 1:**
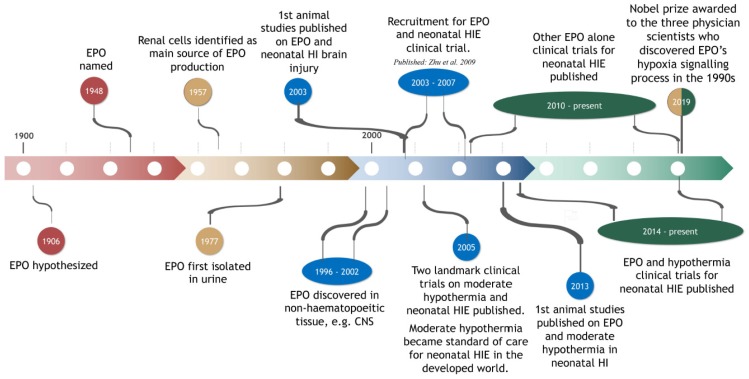
Timeline of the history of EPO in the context of its discovery, hypoxia-induced signalling processes (and the associated Nobel Prize), its use in animal studies on perinatal hypoxia-ischemia (HI) and its use in clinical trials on neonatal hypoxic-ischemic encephalopathy (HIE).

EPO was first hypothesized to exist in 1906 by Carnot and Deflandre [25] (see Figure 1). They reported that plasma from a donor rabbit, that had experienced bleeding, induced a prompt increase in the number of immature red blood cells (i.e., reticulocytes) when injected into a recipient rabbit [26]. The existence of EPO was confirmed over the ensuing decades, although not without substantial controversy (see review by Sytkowski [27]). The term EPO was introduced in 1948 [28]. In 1977, in a landmark study, EPO was isolated from human urine by Goldwasser, Kung and Miyake [29] (Figure 1). This eventually led to the production of human recombinant EPO (r-Hu-EPO [30]) for use in animal research and clinical trials (see Section 3, Section 4, Section 5, Section 6, Section 7 and Section 8), and the production of antibodies to EPO.

EPO’s main role is to prevent apoptosis of erythroid progenitor cells and to enhance their maturation and proliferation [26]. To induce its effect, EPO binds to a homodimeric EPO receptor on the external surface of erythroid progenitor cells [23]. This induces phosphorylation of several tyrosine residues in the intracellular domain of the receptor, which ultimately leads to the activation of prosurvival, proproliferation and prodifferentiation genes in the progenitor cells [31]. EPO is produced in fetal hepatocytes and mainly by the kidney (peritubular cells) in adults [32,33]. These cells release EPO into the bloodstream, which in turn carries the EPO to the bone marrow to act on the erythroid progenitor cells [34].

**Figure 2:**
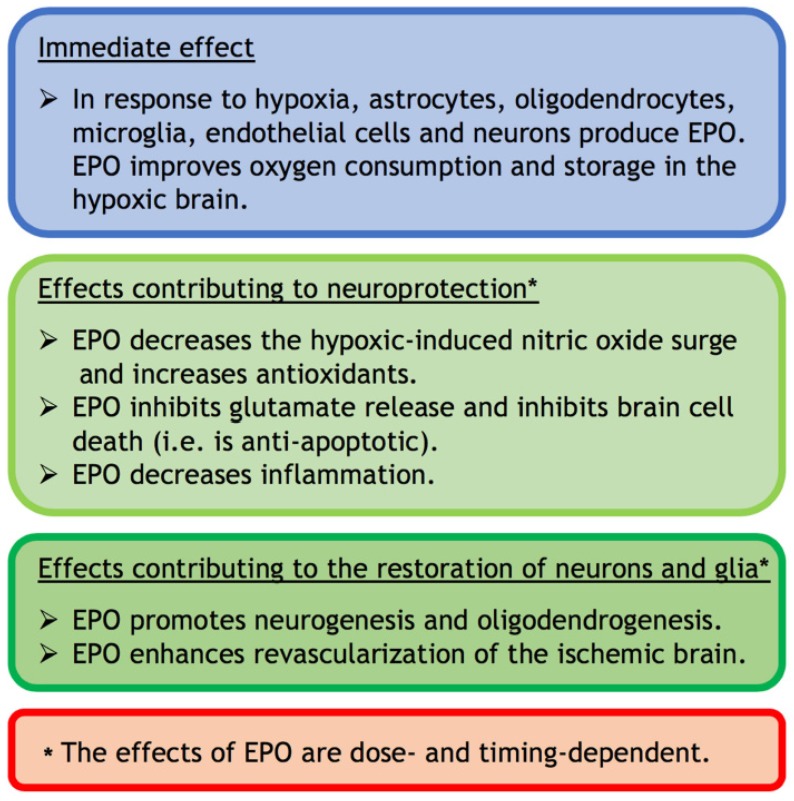
A summary of the immediate, neuroprotective, and restorative effects of EPO in the hypoxic-injured brain.

Initial experiments supporting an action of EPO outside the hematopoietic system, specifically in the nervous system, were published between 1993–2002 [34,35,36,37,38]. This work depended in part on the commercial availability of polyclonal antibodies against the EPO receptor [27]. Expression of EPO and/or its receptor was detected for neurons, astrocytes, endothelial cells, microglia, and oligodendrocytes in the central nervous system of rodents and humans (see Table 1 in ref. [34]; See also refs. [36] and [38]; Figure 2 in this review). All commercially available antibodies of the EPO receptor were, however, hampered by their non-specific cross-reactivities [39]. This called into question their expression in non-hematopoietic tissues. Using new, highly specific polyclonal antibodies specifically directed against different epitopes in the cytoplasmic tail of the mouse and human EPO receptor, Ott et al. 2015 [40] confirmed the expression of the EPO receptor by neurons, astrocytes, microglia and oligodendrocytes and the up-regulation of EPO receptors in response to brain injury.

Within the brain at least three versions of the EPO receptor are now known to exist, including the common beta chain receptor [23]. While the heteromic EPO receptor involving the common beta chain receptor was initially thought to confer neuroprotection, evidence now supports a role for this heteromic EPO receptor *and* the homodimeric EPO receptor in the protection of neurons and glia [23].

In the brain, investigation of the functions of EPO was initially undertaken using cultured cells prior to in vivo studies. EPO is primarily produced by cultured astrocytes as a highly specific neuronal growth factor that is up-regulated by hypoxia [38,41]. Oligodendrocytes, endothelial cells, neurons, and microglia can also produce EPO that is up-regulated by hypoxia [42]. (Figure 2). The oxygen-dependent regulation of EPO is controlled by hypoxia-inducible transcription factor (HIF)-1α. This factor can be expressed by cultured cells within 30 minutes of exposure to hypoxia, which was detected by Nobel Laureate Semenza and his colleague Wang [43]. The two other Nobel Laureates, Kaeilin and Ratcliffe and their respective teams, discovered that HIF degradation was guided by the ubiquitin-ligase von Hippel Lindau protein [44] and that hypoxia made the HIFα stable, through a hydroxylation process, and transcriptionally active [45,46].

Nitric oxide was shown to directly inhibit HIF-1α [47]. In neonatal rats in vivo, EPO significantly reduced the injurious nitric oxide surge that occurs in brain tissue secondary to cerebral ischemia [48] (see also Section 3). It needs to be acknowledged, however, that inhaled nitrous oxide is an effective pulmonary vasodilator in newborns with persistent pulmonary hypertension [49,50]. Hence, in these newborns, the use of EPO to inhibit nitrous oxide production could be detrimental by increasing pulmonary hypertension.

Intrinsic EPO released secondary to hypoxia, or extrinsic EPO administered to treat hypoxia in mice, lead to neuronal expression of hemoglobin which improves oxygen consumption and storage in the hypoxic brain [51]. Since the neonatal brain has a high concentration of unsaturated fatty acids in neuronal membranes, low concentration of antioxidants and high oxygen consumption, it is highly vulnerable to oxidative damage that occurs in HIE [52]. Modulation of the antioxidant defence system by reducing oxidative damage and increasing the antioxidants may contribute to EPO’s neuroprotective potential [53,54] (see also Section 3). EPO can also have a direct antioxidant effect that protects the brain from oxygen free radicals (Figure 2) [53,55,56].

In addition (see Figure 2), EPO can inhibit brain cell death (i.e., is anti-apoptotic [38,57]), protect glial cells [58,59] and spare hippocampal neurons [60]. EPO is thought to achieve these protective functions by inhibiting the exocytotic release of glutamate [61]. Glutamate excitotoxicity is the major cause of the self-sustaining primary destructive cascade in HIE [1,62]. EPO can also decrease inflammation and inflammatory mediators, which play a key role in the latent phase of neuronal damage following HIE [3,62].

Furthermore, EPO can promote neurogenesis [57] and oligodendrogenesis [63] (Figure 2) and enhance revascularization of the ischemic brain [57,64]. The proangiogenic effects of EPO are likely to occur through its interaction with vascular endothelial growth factor and its ability to induce mitosis and migration of endothelial cells [65].

In summary, within the brain EPO has anti-nitric oxide, antioxidant, anti-apoptotic, anti-excitotoxic, and anti-inflammatory functions that can protect neurons as well as glial cells. EPO can also have neurogenic and oligodendrogenic properties, and can improve blood flow, within the injured brain. Hence, EPO can be both neuroprotective and neurorestorative [42]. All these effects of EPO are dose-dependent [56] and timing-dependent (see Section 3; Figure 2). The effects of EPO depend therefore on the context.

With respect to in vivo neonatal hypoxic-ischemic brain injury, the evidence for these functions of EPO is discussed in more detail in the next section. In terms of pathophysiology, primary energy failure occurs at the time of the hypoxic-ischemic insult, with secondary energy failure occurring at 24–48 h [66]. The secondary energy failure heralds the onset of delayed neuronal necrosis [66]. Hence, EPO treatment commenced within 24 h of the insult could potentially be effective.

## 3. Animal Studies on Neonatal Hypoxia-Ischemia: Effect of EPO Alone

EPO has been studied in neonatal animals in which brain hypoxia-ischemia was induced to simulate conditions affecting newborns suffering from HIE. Most of these studies have used r-Hu-EPO since EPO from humans was the first to be purified and cloned [30] (see Table 1). r-Hu-EPO is derived from cultured mammalian hamster ovary cells at high levels of purification and in sufficient quantities, due to difficulties in obtaining enough purified EPO from the blood of living humans [30,67]. Forty percent of EPO consists of sugar (i.e., carbohydrate) chains that are essential for its activity, stability, and biosynthesis [67]. There are at least nine known isoforms of r-Hu-EPO including epoetin alfa/alpha/beta, darbepoetin and carbamylated EPO (see Table 1 in ref. [68]). The EPO analog darbepoetin contains additional sialic acid (i.e., additional sugar) which confers a three-fold longer serum half-life compared to the more commonly used epoetin alfa [69]. Epoetin alfa has been used extensively in clinical trials (see Section 5 and Section 6). Carbamylated EPO, in which all lysines are transformed to homocitrulline, is not erythropoietic [70]. In the subsequent sections on animal and clinical research, most studies have used r-Hu-EPO although the EPO isoform used is generally not indicated. When the specific isoform is indicated, we have stated this.

In 2003 the first studies on the use of EPO to protect the developing brain against neonatal hypoxia-ischemia were undertaken on postnatal day (PN) 7 mice using EPO [71] or rats using r-Hu-EPO [72,73] (see Table 1). PN7 rats and mice were used because their brain at this age is generally considered to be equivalent to the developmental stage of the human brain during the late third trimester [74,75]. The well characterized Rice-Vannucci rodent model of neonatal hypoxic-ischemic brain injury [76] was used in all three studies and in most of the subsequent studies reviewed in this section. The model involves the ligation of one of the common carotid arteries followed by exposure to systemic hypoxia. This leads to permanent damage in the cerebral hemisphere that is ipsilateral to the ligated artery. This animal model has reasonable face validity in that it yields behavioural and biological outcomes similar to the human context. It also has reasonable construct validity in that the biological mechanisms in the brain have many similarities to the human context in terms of blood flow changes and cellular metabolic derangements [77]. The unilateral rather than global insult, and the lack of dysfunction across many organs, needs to be acknowledged for this animal model [77,78].

The three initial studies in mice or rats reported that treatment with a single dose of EPO, either 1 h before or immediately after hypoxia-ischemia, significantly reduced the cerebral infarct volume or the cerebral injury score at 1–7 days post-injury compared to hypoxic-ischemic saline-treated animals [71,72,73]. The dose of EPO and the route of administration used for these studies [71,72,73], and all other studies in this section, is indicated in Table 1. Supraphysiological doses were used intraperitoneally [71,73], rather than the doses appropriate for erythropoiesis (i.e., 200–400 U/kg), since a landmark study showed that higher doses enable a sufficient quantity of EPO to cross the blood-brain barrier to provide neuroprotection in the adult brain [56,79].

Subsequent studies in 2004 and 2005 used neonatal hypoxic-ischemic rats to evaluate the effects of immediate post-treatment with EPO on potential mechanisms for the observed neuroprotection, as well as long-term histological and behavioural outcomes. Interestingly, it was noted for the first time that neonatal hypoxia-ischemia dramatically up-regulated the expression of EPO receptors in the brain at 24 h post-injury [80,81]. r-Hu-EPO delivered immediately after PN7 hypoxia-ischemia significantly decreased the overproduction of cerebral nitric oxide at 72 h post-injury [48]. It also decreased the extent of lipid peroxidation and increased the activity of the antioxidant enzyme glutathione peroxidase at 24 h post-injury [53]. Thus, mechanisms by which EPO could be neuroprotective in neonatal hypoxia-ischemia were identified.

In longer term experiments, r-Hu-EPO delivered immediately after PN7 rat hypoxia-ischemia significantly improved long-term memory on a water maze, as well as the residual cerebral volume, at 20 weeks post-injury [60]. McClure et al. [82] confirmed and extended these positive effects of EPO on memory in 2–3-month-old hypoxic-ischemic rats. They also demonstrated a sparing of rapid auditory processing after this treatment with EPO. However, more recent evidence showed little or no therapeutic benefit for auditory processing after a 1 h or 3 h delay in the onset of treatment with EPO [83]. EPO delivered immediately after PN7 rat hypoxia-ischemia significantly improved recovery of sensorimotor function, and reduced brain damage, at 6 weeks post-injury [84]. While these functional and anatomical outcomes showed significant long-term improvement after immediate post-injury treatment with EPO, it is noteworthy that the rescue was generally not to normal control levels.

Repeated daily dosing, for 3 days post-hypoxic-ischemic injury in PN7 rats, prevented amphetamine-induced rotational asymmetry and reduced sensory neglect 3 weeks later [85]. However, it did not prevent sensory neglect altogether. In addition, this treatment strategy did not significantly affect the overall brain injury score [85]. This may be because the hypoxic insult was milder at 1.5 h compared to 2.5 h in the earlier study by Kumral et al. [73]. It may also be due to the timing of the EPO treatment since it is unclear if the post-treatment by Demers et al. [85] started immediately after hypoxia-ischemia as in Kumral et al. [73].

A subsequent 2009 study also reported that EPO was not significantly neuroprotective of cerebral grey or white matter damage. Specifically, they also used a milder form of hypoxia of 1.5 h and post-treatment with r-Hu-EPO (epoetin beta) in PN7 rats [86]. These results led to an investigation that included an assessment of EPO on neural repair in the brain, specifically the effect on progenitor cells in the subventricular zone and dentate gyrus after mild hypoxic injury of 45 minutes [87]. Post-treatment with EPO in PN9 mice significantly increased the number of progenitor cells 72 h later, although this was not correlated with improved functional outcomes [87]. The increased number of progenitor cells occurred in females but not males. In the preceding studies discussed in this section, most did not specify the sex of the PN7 pups used or indicated that pups from both sexes were used. McClure et al. [82] specifically used male pups. Hence a gender effect [87] for high dose EPO treatment after milder hypoxic injury in neonatal hypoxic-ischemic pups was a novel finding.

A gender effect for higher dose EPO treatment (see Table 1) had been reported earlier for neonatal stroke [88]. Specifically, PN7 rat pups exposed to permanent focal vascular cerebral injury involving the middle cerebral artery, followed by EPO treatment, showed a significant long-term neuroprotective effect, with this effect more beneficial in females [88]. Possible explanations for this difference include that gender modulates responsiveness to r-Hu-EPO in the kidney [98] and EPO receptor alleles are present at markedly higher frequency in females than males [99]. In addition, the cell death pathways in males and females are different, which might explain the different effects of EPO in male/female animals [100]. Taken together, these findings highlight that the effect of EPO is likely to be dependent on the severity of a brain injury and on gender. Future studies require consideration of both contexts.

It is evident from the study of Wen et al. [88] that the effects of EPO have also been investigated in neonatal animal models of focal stroke. Single- and multiple-dose treatment regimes of r-Hu-EPO after neonatal focal ischemic stroke in rats have reduced the cerebral infarct volume in a dose-dependent manner [89] and have led to short-term improvements in sensorimotor outcomes [90]. In longer term experiments, rats treated with three doses of r-Hu-EPO (see Table 1) after neonatal stroke did not differ from shams in memory performance at 3 months-of-age [91]. The same treatment regime for r-Hu-EPO also increased neurogenesis and oligodendrogenesis in the subventricular zone after rat neonatal stroke [92]. Hence neonatal models in mice and rats of focal stroke, and of hypoxic-ischemic brain injury, report neuroprotective and/or neurorestorative effects of treatment when EPO was administered in a single dose before injury or commenced immediately after injury using one or multiple doses of EPO. A comparison of a range of doses and treatment frequencies showed that for treatment immediately after injury in PN7 hypoxic-ischemic rats, three daily doses of r-Hu-EPO (Procrit, apoetin alfa) at 5000 U/kg was optimal because it provided maximal benefit against brain injury at 24 h and 7 days post-injury, with limited total exposure to EPO [93].

The more clinically relevant effect of delayed treatment with EPO was also investigated in the PN7 neonatal hypoxic-ischemic rat (see Table 1). Delayed treatment with r-Hu-EPO decreased the cerebral infarct volume 1 week later and improved motor outcomes 4 weeks later [94]. Delayed treatment with r-Hu-EPO, commencing at 1 day after PN7 rat hypoxia-ischemia was also neuroprotective, prevented the delayed secondary rise in interleukin 1-beta, and attenuated the infiltration of leukocytes at 2 weeks post-injury (see Table 1) [95]. When the onset of treatment with r-Hu-EPO started at 2 days post-hypoxic-ischemic injury in PN7 rats, with further treatment on 4 days until day 13, EPO was ineffective in preventing brain volume loss but significantly improved oligodendrogenesis and sensorimotor function at the extended period of 14 days post-injury [96]. These results indicate that repair mechanisms triggered by EPO treatment may require time to be significantly effective, and that delayed treatment with EPO is effective in neonatal hypoxic-ischemic rats.

Nanomedicine can enhance brain drug delivery by up to 50 times [101], bypassing usual blood-brain barrier routes, as well as avoiding the possibility of a thrombotic risk of high dose EPO due to its erythropoietic function [97]. Using a nano form of EPO, it was found that 300 U/kg nano EPO (PLGA-EPO-NP) had a neuroprotective effect comparable to 5000 U/kg rEPO at 72 h post-injury [97]. The nano EPO particles were given as intraperitoneal injections, at 1 h, 24 h and 48 h post-injury, in PN10 neonatal hypoxic-ischemic rats. Functional deficits were found to be significantly reduced in the 300 U/kg nano EPO group when compared to vehicle controls. The deficit attenuation was similar in the nano EPO group compared to a 5000 U/kg r-Hu-EPO-treated group. Thus, the nano form of EPO yielded the same result at a much lower dose.

In summary (Table 1), neonatal studies in mice and rats have shown that early administration of a single dose, or repeated doses, of EPO at 1000–5000 U/kg is both neuroprotective and neurorestorative. Early administration of a nano form of EPO is just as effective as rEPO and can be administered in a much smaller daily dose. Less brain damage and improved behaviour is also evident after delayed repeated treatment with r-Hu-EPO. Of note, the improvements seen after treatment with EPO alone have generally not been to the level of control, uninjured animals. This suggests that combining EPO treatment with an adjunct therapeutic strategy should be researched. In the next section, more recent studies that have investigated a possible additive effect of the combination of EPO with moderate HT are discussed. Moderate HT alone is neuroprotective after neonatal hypoxia-ischemia in animals [102]. Moderate HT also reduced the degree of disability after neonatal HIE in two landmark randomized clinical trials published in 2005 [103,104] (see Figure 1).

## 4. Animal Studies on Neonatal Hypoxia-Ischemia: Effect of EPO in Combination with Moderate Hypothermia

In 2013 (see Table 2), the first three animal studies were published on the combined effects of EPO and moderate hypothermia after neonatal hypoxia-ischemia during the third trimester equivalent or around term.

Fan et al. [105] investigated motor and histological outcomes after combined treatment with HT and EPO in the Rice-Vannucci PN7 neonatal rat model of hypoxia-ischemia. They exposed the rat pups to hypoxia for 1.5 h, started the hypothermia (32.5–33 °C for 3 h) immediately after hypoxia, and administered r-Hu-EPO (Eprex EPO, epoetin alfa) immediately after hypothermia and at 24 h and 48 h. Motor skills were tested at 2 and 5 weeks post-injury and were impaired for the untreated normothermic/saline group. HT alone, and HT plus EPO, improved motor skills, but this improvement was only mild for the EPO alone group. Interestingly, there appears to be no previous PN7 hypoxic-ischemic rat study on the effects of EPO alone at the specific dose used (5000 U/kg) and treatment onset and frequency. The positive effect of HT plus EPO was not greater than the effect of HT alone. The histological outcome at 5 weeks was only improved by treatment with hypothermia alone, with a more pronounced effect in females. Adding EPO to HT had only a borderline neuroprotective effect at 6 weeks (*p* = 0.07). The possibility that EPO alone, and the combination of EPO and HT, is more effective in milder models of hypoxia-ischemia could not be excluded [105].

Fang et al. [106] also investigated motor and histological outcomes after combined treatment with HT and EPO in the Rice-Vannucci PN7 neonatal rat model of hypoxia-ischemia. They exposed the rat pups to a longer period of hypoxia (2 h), delayed the treatment with hypothermia (31 °C for 8 h) to start from 1 h after hypoxia, and administered r-Hu-EPO immediately after hypoxia and at 24 h and 7 days. Of note, deeper hypothermia at 31 °C was used and the control animals were hypothermic too at an average of 33.8 °C for 8 h. Motor skills were tested at 2 and 6 weeks post-injury, but importantly they were unexpectedly not impaired for the untreated normothermic/saline group thereby confounding comparisons. There was also a large variation in all groups for the pathological outcomes, no correlation between the behavioural and histological results, and neither EPO alone nor hypothermia alone nor the combination were effective. Treatment with EPO alone appeared to be beneficial in males, although the graph of the linear regression analysis is not provided nor the relevant sample sizes. Due to the aforementioned confounding factors, the most useful outcome from this study was that there were no adverse effects with the combined treatment.

Using a term non-human primate model of perinatal asphyxia, Traudt et al. [107] investigated the effect of combined EPO and HT treatment in the long-term (i.e., up to 9 months-of-age, which is comparable to 3 years of human development). After 15–18 minutes of umbilical cord occlusion in *Macaca nemestrina* at 1–8 days prior to term, animals received either saline, or HT alone, or HT plus EPO. An EPO alone group was excluded due to low animal numbers for the final analyses. HT of 33.5 °C for 72 h commenced after resuscitation of an infant and always commenced by the 3rd hour of life. rEPO (epoetin alfa) was given at four time points post-hypoxia and at various doses (see Table 2). The major findings of the study were that, while HT alone did not reduce the risk of death or moderate-severe cerebral palsy after umbilical cord occlusion, HT combined with four doses of EPO significantly reduced this risk to 0%. The risk of death or moderate-severe cerebral palsy was 43% after treatment with saline. HT plus EPO also preserved normal motor functions and preserved cerebellar growth. Cerebellar growth was also improved by treatment with HT alone. Two strengths of this study were that the animal model used produced very similar physiological and neurological outcomes to human infants with moderate to severe HI and that there were no adverse events due to combined treatment with HT and EPO. Significantly, the positive outcomes for HT combined with EPO in this non-human primate study formed the basis for initiating clinical trials in human neonates (see the next section).

In a follow-on study in 2017 by McAdams et al. [108], the same groups and treatment regimes as Traudt et al. [107] were used. None of the animals treated with combined HT and EPO demonstrated signs of long-term neuropathological toxicity at 9 months-of-age. This supports further research of this combined strategy to promote improved long-term outcomes after neonatal HIE.

## 5. Clinical Studies on Neonatal Hypoxic-Ischemic Encephalopathy: Effect of EPO Alone

Due to EPO’s effects on the production of red blood cells, it has been used clinically since 1990 to treat anemia of prematurity [109,110]. The use of EPO reduces the need for blood transfusions in premature infants. To cross the blood-brain barrier, high doses at 2000 to 5000 IU/kg body weight are administered either early or late for prolonged periods of time. These high doses are well tolerated in preterm infants (i.e., EPO is safe and devoid of untoward complications in this context [111,112,113]).

Based on the results of animal studies (e.g., Kumral et al. [70], see Section 3), the first human study by Zhu et al. 2009 [114] used EPO in the treatment of HIE recruited 167 term newborn infants born between August 2003 and January 2007 (see Figure 1, Table 3). The neonates had moderate to severe HIE and were treated either with conventional treatment (*n* = 84) or a low dose of r-Hu-EPO (*n* = 83). Conventional treatment in most neonatal centers includes respiratory support, fluid infusion, anti-convulsants, reducing intracranial pressure, ionotropic support to maintain blood pressure and the correction of hypoglycemia, acidosis, and electrolyte imbalance [115]. The first dose of r-Hu-EPO was administered at 1 to 48 h after birth, followed by doses every other day for 2 weeks. At 18 months-of-age neurodevelopmental outcomes were assessed. Improved long-term outcomes in the r-Hu-EPO-treated infants were evident after moderate HIE, but not in those with severe HIE. There were no side-effects from r-Hu-EPO treatment [114].

Elmadhy et al. 2010 [116] (Table 3) recruited 45 neonates and divided them into three groups: (a) 15 normal infants, (b) 15 infants affected with HIE and receiving only conventional treatment (control) and (c) 15 infants affected by HIE who were treated by conventional treatment plus five daily doses of subcutaneous r-Hu-EPO, with the first dose given within 4–6 h after birth followed by four daily doses [116]. The study compared the serum concentration of nitric oxide, electroencephalograms (EEGs), magnetic resonance imaging (MRI) of the brain, and neurologic and developmental outcomes of the three groups. The two HIE groups had a significantly higher nitric oxide concentration at enrolment compared to healthy neonates. This concentration was significantly reduced in the r-Hu-EPO group after 2 weeks compared to the HIE control group. The incidence of breakthrough seizures was also significantly decreased in the r-Hu-EPO group after 2 weeks compared to the HIE control. However, the MRI changes after 3 weeks were similar in the r-Hu-EPO and HIE control groups. For the neurodevelopmental assessment, the Denver II screening test was used [120]. This assessment at 6 months found significantly fewer neurologic and developmental abnormalities in the r-Hu-EPO group compared to controls [116].

Avasiloaiei et al. 2013 [117] (Table 3) compared three groups of term neonates affected by HIE: 22 neonates were treated with EPO for the first 3 days plus supportive care, 22 neonates were treated with phenobarbital during the first 4 h after birth, and 23 neonates received supportive care alone as the control group. The neurodevelopmental delay at 18 months-of-age was lower in both of the treated groups compared with the control group, although the differences were not statistically analyzed. El Shimi et al. 2014 [118] (Table 3) compared 30 term neonates with HIE with 15 healthy neonates. Ten HIE neonates were treated with a single dose of r-Hu-EPO on day 1, 10 were treated with therapeutic hypothermia for 72 h, and 10 with supportive care. At 3 months-of-age, there were no significant differences between the groups in terms of their neuromuscular function or their brain score after MRI. A limitation for both studies is the relatively small sample size, and the use of a single dose of r-Hu-EPO in the latter study [121].

In a 2017 study by Malla et al. [119] (Table 3), 100 neonates with moderate or severe HIE were recruited to a randomized study investigating the effectiveness and long-term outcomes of repeated doses of EPO alone, when HT is unavailable (e.g., in resource poor settings). Neonates were randomly assigned to treatment or placebo (50 per group). Neonates received a total of five doses of either r-Hu-EPO, or 2 mL of saline (placebo group), given on alternate days. Treatment (EPO or saline injections) was started within 6 h of birth. Neonates were monitored until death or up to approximately 19 months-of-age. Death occurred in 16% of neonates across both groups at 19 months-of-age. The difference in each of the following outcomes was statistically significant. Death or disability occurred in 70% of neonates in the placebo group compared to 40% of neonates in the EPO-treated group at 19 months-of-age. The EPO-treated group had a lower risk of cerebral palsy and showed less neurological abnormalities using MRI. The study concluded that repeated doses of r-Hu-EPO, started within the first 6 h after birth, improved outcomes (i.e., reduced death and disability) in neonates. Hence, monotherapy with repeated doses of EPO, starting within 6 h, is effective in resource poor situations where HT is unavailable [119].

The promising findings from three of these clinical trials on the effect of EPO monotherapy on neonatal HIE [114,116,119] now need to be investigated in a large, multicentred, randomized controlled trial that reports long-term neurological outcomes.

## 6. Clinical Studies on Neonatal Hypoxic-Ischemic Encephalopathy: Effect of EPO Combined with Moderate Hypothermia

Based on the findings of Traudt et al. [107] in 2013 in the neonatal hypoxic *Macaca nemestrina* (see Section 4), a number of clinical trials have been recently undertaken to assess combination treatment using HT and EPO. A Phase I/II study by Baserga et al. [122] (Table 4) used standard body (primarily) or head cooling commenced at less than or equal to 11 hours of age in addition to darbepoietin. The darbepoetin was administered within 11 h of birth, with a second dose 7 days later. This is known as the Darbe Administration in Newborns Undergoing Cooling for Encephalopathy (DANCE) study. As indicated earlier in Section 3, darbepoetin is an engineered form (i.e., analog) of EPO that contains additional sialic acid. This confers a three-fold longer serum half-life compared to the more commonly used (see below) epoetin alfa [69]. This study proved the safety of using HT plus EPO in neonates and showed that the pharmacokinetics was sufficient for weekly administration of EPO [122].

Valera et al. [123] (Table 4) treated 15 HIE neonates with EPO every 48 h for 2 weeks, commencing within 3 h of birth along with therapeutic hypothermia for 72 h. At 18 months, there was 80% survival with no neurodevelopmental disability. Unfortunately, there was no control group for comparison.

Rogers et al. [124] (Table 4) recruited 24 newborns with HIE and administered up to six doses of r-Hu-EPO (Procrit or epoetin alfa, based on the clinical trial number NCT 00719407). The EPO treatment was started 24 h after HIE and then given every 48 h, and was combined with standard HT (33.5 °C for 72 h within 6 h of HI) [124]. Outcomes were available for 22 of the 24 infants. The authors found that significant neurodevelopmental disability occurred in only 12.5% of infants with moderate to severe MRI changes who received HT plus EPO, compared to 70%–80% significant disability or death occurring in infants treated with HT only as part of the National Institute of Child Health and Human Development hypothermia trial. Both groups had comparable moderate to severe brain injury using MRI [124]. However, these results were not statistically significant as the number of infants was too small. Hence, further studies were warranted to determine whether HT and EPO could improve outcomes at a statistically significant level.

A Neonatal Erythropoietin and Therapeutic Hypothermia Outcomes (NEATO) Phase II study by Mulkey et al. 2017 [125] (Table 4) was carried out in seven US centers on 50 newborn infants with moderate to severe HIE. Infants were randomized to receive either (a) five doses of EPO, or (b) an equal volume of normal saline (placebo). The doses were administered on days 1, 2, 3, 5 and 7-of-age. Both groups were treated with HT either of the whole body or by head cooling at 6 h after birth to the universally accepted standard of 33.5 °C for 72 h. The first dose of r-Hu-EPO (Procrit or epoetin alfa, based on the clinical trial number NCT 01913340) was given less than 24 h after birth. The use of MRI to detect ischemic brain injury in 44 infants showed that, among the 20 infants with acute brain injury detected before 7 days-of-age, those receiving HT plus EPO had a statistically significant lower volume of acute brain injury compared to HT-saline-treated infants [118].

As part of the NEATO Phase II study, Wu et al. [126] (Table 4) studied the 50 newborns treated with HT for moderate/severe HIE. The newborns who received HT as a standard routine measure for HIE were randomized to either additional EPO or placebo. The study showed that 24 infants who were given a high dose of r-hu-EPO on days 1, 2, 3, 5 and 7, plus HT, had significantly less brain injury at 5 days-of-age (on MRI) and better 12-month motor outcomes compared to the 26 infants given placebo [126]. The first dose of r-Hu-EPO (Procrit or epoetin alfa, based on the clinical trial number NCT 01913340) was given on day 1 at an average age of 16.5 h. Phase I of the study was carried out earlier on neonates with HIE and proved that the optimal neuroprotection plus safety from side-effects could be achieved by five intravenous doses of r-Hu-EPO 1000 U/kg/dose administered between days 1 and 9-of-age [127]. Preliminary rat studies determined the optimal neuroprotective dose of recombinant EPO (rEPO) used in the Phase I trial based on peak concentrations of plasma r-Hu-EPO in brain tissue [128].

As part of the NEATO Phase II study, Wu et al. [129] correlated the placental pathology with the brain MRI scores of the 50 newborns treated with HT and EPO, or HT and placebo, for moderate/severe HIE. Among subjects with no chronic placental abnormality, treatment with EPO was associated with a lower global brain injury score and a lower rate of subcortical injury. This was not the case for patients that had a chronic abnormality in the placenta. Hence the placenta may provide a guide to treatment response in HIE.

Based on these findings, a Phase III multicentred High-Dose Erythropoietin for Asphyxia and Encephalopathy (HEAL) study, by Wu, Juul and colleagues [130] (Table 4) is recruiting 500 newborns ≥36 weeks-of-age with moderate/severe HIE. Infants will be treated with a 1000 U/kg intravenous EPO on study days 1, 2, 3, 4 and 7. The 1st dose of r-Hu-EPO (Procrit or epoetin alfa, based on the clinical trial number NCT 01913340) is planned to be given within the first 24 h postnatally. Assessment of brain damage by MRI and MRS (Magnetic Resonance Spectroscopy) is planned between days 4 and 6-of-age. Developmental assessment will be achieved through parental telephone interviews at 4, 8, 12, 18 and 24 months-of-age. The areas covered by the assessment will be: gross motor function, cognitive abilities and language, presence of cerebral palsy, presence of epilepsy and/or any behavioural abnormalities (especially those related to attention and aggression). The aim is to determine if high dose EPO in combination with moderate hypothermia reduces mortality and neurodevelopmental disabilities in term infants with moderate to severe HIE [130]. A similar Phase III trial is underway in Australia, the Preventing Adverse Outcomes of Neonatal Hypoxic Ischemic Encephalopathy with Erythropoietin (PAEAN) trial involving 300 neonates (NCT03079167) [131].

A Phase III study (NEUREPO, clinical trial number NCT01732146) by Patkai et al. [132] (Table 4) is ongoing. Patkai et al. are using standard HT within 6 h of birth plus beta r-Hu-EPO. The beta subtype is another isoform of r-Hu-EPO. While it differs from the alfa isoform in its glycosylation, pharmacokinetics and pharmacodynamics, it has the same clinical efficacy as epoetin alfa in hemodialysis patients [133]. The first dose of beta r-Hu-EPO is given at <12 h, and the second and third doses are administered every 24 h thereafter. Their primary outcome measure will be survival without neurological sequelae at 24 months-of-age [132]. The publication of these results is anticipated to be in the fall (for the northern hemisphere) of 2020 (Patkai, personal communication, January 2020).

In a 2017 study by Wang [134] (Table 4), 68 newborn infants affected by HIE were randomized into two equal groups: (a) 34 infants (control) received conventional treatment plus ascorbic acid (Vit C) once daily plus r-Hu-EPO, both intravenously, three times a week (with the commencement time of treatment with the r-Hu-EPO not stated); (b) 34 infants were given a similar treatment (Vit C and r-Hu-EPO) plus moderate hypothermia using water cushioning of the body to maintain the rectal temperature at 33.5 °C for 72 h 131]. The two groups were compared before treatment, and 3 and 7 days after treatment, regarding: (1) Target organ injury markers. These were significantly reduced *after* treatment in both groups, with some markers lower after treatment with Vit C, HT and r-Hu-EPO (i.e., they were even lower in group b); (2) Oxidative stress index levels. These were lower, and antioxidant enzymes were higher, in both groups after treatment, with better results again in group b; (3) Apoptosis index levels. These showed lower pro-apoptotic molecules, and higher anti-apoptotic molecules, in both groups after treatment, with better results again in group b. All the above parameters were the same before treatment [134]. Although the study did not state whether asphyxia was mild, moderate or severe, the author concluded that combining moderate hypothermia with r-Hu-EPO (plus vitamin C) provided the best protection from the damage resulting from neonatal asphyxia, and this protective effect was achieved through inhibiting apoptosis, reducing oxygen free radicals and enhancing antioxidant capacity [134].

Published in 2019, Nonomura et al. in Osaka, Japan studied combined therapy to treat nine neonates with moderate to severe HIE using EPO, magnesium sulphate and HT [135] (Table 4). All neonates were treated within 6 h of birth with whole body cooling at 33.5 °C for 72 h and epoietin alfa was given every other day, for 2 weeks. Magnesium sulphate was also given within 6 h of birth in three daily doses. Magnesium sulphate was used because it reduces glutamate-mediated excitotoxicity [136]. There were no deaths and all nine neonates did not have any serious adverse events [135].

There are also other neuroprotective agents (see, for example, ref. [137]) that could be investigated in combination with EPO and HT in future studies.

It is evident, most importantly, from these current human studies that the effectiveness of combined treatment with moderate HT and EPO for neonatal HIE looks promising, yet awaits the outcomes of current clinical trials on neurological outcomes at 18–24 months-of-age. If these results are promising, a greater number of infants in more randomized controlled trials across multiple centres would be required before this treatment could be considered for routine clinical use. Longer term follow-up will also be necessary to determine whether combined treatment with moderate hypothermia and EPO for neonatal HIE sustained effectiveness on neurological outcomes.

It is also evident (Table 3 and Table 4) that the route used for administering r-Hu-EPO for HIE in human neonates is subcutaneous or intravenous. There is also variability in dosage (ranging from 300 U/kg up to 2500 U/kg), the starting point (at 4–6 h post HIE or delayed up to 48 hours after birth), as well as the frequency of injections (daily, alternate days) and length of treatment (four doses or up to 12 doses). Hence, the most effective dose, starting point, frequency and length of treatment remains to be determined.

Interestingly, all parameters measured by Wang [134] showed improved short-term biochemical and histological outcomes for neonatal HIE infants treated by combining r-Hu-EPO, Vit C and hypothermia compared to r-Hu-EPO and Vit C alone. Since another combined treatment (i.e., EPO plus magnesium sulphate plus HT) yielded no adverse events [135], more triple combined treatments could be investigated in animals and in humans to determine if greater neuroprotection and less disability can be achieved when compared with the dual combined treatment of EPO and HT.

## 7. Effects of EPO in Clinical Trials for Preterm Infants

The detection of EPO receptors in the brain (e.g., ref. [37]) led to rodent studies that showed that EPO rescues neural cells after brain hypoxia in the preterm-equivalent. For example, Mazur et al. 2010 [138] reported the rescue of neural cells in PN5 and PN9 rats that were exposed to transient systemic hypoxia-ischemia on embryonic day 18 and then treated with r-Hu-EPO (1000 IU/kg daily) over PN 1–3. These findings led in turn to clinical trials (see below). Subsequent studies in preterm lambs suggested that a lower single dose of EPO at 1000 IU/kg may be the optimal dose, compared with 3000 IU/kg and 5000 IU/kg, to achieve both lung and brain protection in preterm infants [139,140,141,142]. The latter results are particularly relevant to ventilated preterm infants, which constitute the majority of these neonates.

In preterm infants born at 27–28 weeks-of-gestation, retrospective analysis of patients treated with EPO for anemia showed that EPO-treated infants had improved neurodevelopmental outcomes [143,144]. Treatment with r-Hu-EPO of babies born at 29 weeks-of-gestation also yielded fewer infants with abnormal scores for white matter injury and grey matter injury, when compared to untreated controls, at term-equivalent age [145]. Natalucci et al. [113] evaluated 448 preterm infants born between 26 and 32 weeks-of-gestation, with 228 randomized to treatment with r-Hu-EPO and 220 given placebo. In contrast to previous studies, they found no statistically significant differences in neurodevelopmental outcomes. Of note, Natalucci et al. [113] administered three doses of 3000 IU/kg EPO within 42 h of birth. This high dose of EPO differs from the other studies on neurodevelopmental outcomes in which EPO was administered at 750 to 1500 IU/kg per week [143] or 1200 IU/kg per week (i.e., 400 IU/kg given three times per week [144]). This suggests that the dose of EPO may be important (see also below). In a recent meta-analysis by Fischer et al. [146] of four randomized control trials, including Natalucci et al. [110], the neurodevelopmental outcome was analyzed for 547 placebo-treated and 574 r-Hu-EPO-treated premature infants (mainly aged 27 to 30 weeks-of-gestation at birth). The results indicated that prophylactic r-Hu-EPO significantly improved cognitive development and had no significant effect on other neurodevelopmental outcomes at a corrected age of 18 to 24 months. These findings demonstrate the effect of statistical power and the promising potential of treatment with r-Hu-EPO for an improved neurological outcome in preterm infants born at 27 to 30 weeks-of-gestation [146].

For extremely preterm infants born at 24 to 27 weeks-of-gestation, Juul et al. 2020 [147] published a Phase III **P**reterm **E**PO **N**e**u**ropro**t**ection (PENUT) clinical trial [NCT01378273] involving 936 subjects. It aimed to evaluate the effect of treatment with EPO on the combined effect of death or severe neurodevelopmental impairment at 24 months-of-age. EPO was delivered within 24 h of birth, involving 6 doses of 1000 IU/kg every 48 h, followed by subcutaneous EPO at 400 IU/kg three times per week up to 32 6/7 weeks post-menstrual age. It was found that treatment with EPO did not result in a lower risk of severe neurodevelopmental outcome or death at 2-years-of-age. The younger age of these preterm infants may have contributed to this outcome [147]. For example, in the studies in the meta-analysis by Fischer et al. [146], the infants were on average more mature at 27 to 30 weeks-of-gestational age. Also, in Fischer et al.’s [146] study, a subset of 117 infants born before 28 weeks-of-gestation did not have significantly improved cognitive development after prophylactic r-Hu-EPO (*p* = 0.18, see their Figure 2B). It is speculated that the contributing factors to neurologic dysfunction are heterogeneous and that the targets that are responsive to EPO may be diluted by pathways not affected by EPO, particularly in the most premature infants [147]. Hence, current data suggests that treatment with EPO may not improve cognition in extremely premature infants.

The higher total EPO dose used in the first week of the PENUT trial may have also contributed to the outcome of no improvement for extremely premature infants [147]. Consideration of all the foregoing results suggests that a single dose of 1000 IU/kg, or multiple doses in a week that do not exceed a total of 1500 IU/kg, may yield an improvement in neurodevelopmental outcomes. These lower doses now need to be evaluated in a large clinical trial on extremely premature infants.

It is also possible that longer term follow-up of the PENUT cohort is needed to identify cognitive and physical problems, and effects of EPO, that may not become apparent until later in life [147].

In summary, these studies highlight the promising neurological outcomes for EPO in preterm infants born at 27 to 30 weeks-of-gestation. When combined with the current promising results for EPO treatment in term infants (see Section 5, Table 3), versus the current results for extremely premature infants described above, the promising effects of EPO may be restricted to infants born in the third trimester and at term. Future clinical trials should clarify this.

## 8. Potential for Adverse Effects of EPO in Neonatal HIE

The potential for adverse effects of EPO during development is-based in part on its induction of the proliferation of neuronal stem cells and its influence on apoptosis [65]. Yet, many premature infants have been treated with EPO for anemia without evident side-effects on brain development. An EPO-induced high hematocrit can also cause brain injury [34]. Yet babies with HIE commonly have lower hematocrits from perinatal events such as placental abruption or from frequent blood sampling for clinical monitoring. In the clinical trials published from 2009–2019 that are listed in Table 3 and Table 4, those that evaluated possible undesired side-effects from EPO treatment found no difference in adverse outcomes or complications, including for the hematocrit, compared to controls [114,116,118,119,122,123,124,127,135].

## 9. Conclusions

For treatment with EPO alone, and for combined treatment with EPO and HT, animal studies and Phase I/II clinical trials showed promising outcomes after neonatal hypoxic-ischemic brain injury during the third trimester or at term. For example, combining HT and EPO after neonatal HIE yielded improved motor outcomes at 12 months-of-age compared with HT alone [126]. The outcome of further clinical studies on disability and functional outcomes at 18–24 months-of-age [130,132] and older ages is awaited because these ages are more informative for later neurodevelopmental outcome. It is thus too early to start using hypothermia plus EPO treatment routinely in human neonates with HIE.

With respect to whether combined therapy with EPO and HT is more advantageous than EPO alone during the third trimester or at term, there are only a few studies thus far that have made this direct comparison. This is due to technical constraints in animal studies (e.g., ref [107] in Section 4) and because the main aim in clinical studies has been to compare combined EPO and HT therapy with the current standard of care of HT alone (see Section 6, Table 4). For one of these comparative studies, a possible advantageous effect of combined treatment in the neonatal rat is of borderline statistical significance (see Table 2 for Ref. [105] and Section 4). For a clinical study, ascorbic acid (Vitamin C) has been included in each group (see Table 4 for Ref [134] and Section 6). This adds a confound to the desired comparison. Thus, whether combined therapy with hypothermia and EPO is advantageous, or not, over treatment with EPO alone requires ongoing research.

Regarding the efficacy of immediate and delayed treatment with EPO, treatment with EPO alone in animal studies showed that both strategies are effective (see Section 3). In animal studies on combined therapy with HT plus EPO, only treatment with EPO within 24 h of hypoxia was studied and this is effective (e.g., Refs. 105,107, Section 4). In clinical studies, it is evident from Section 5 and Section 6 that treatment with EPO within 24 h, and up to 48 h of birth, is effective and that most studies have started treatment within 24 h. The timing of onset in the clinical study by Wang {134} is unknown, but would be within 72 h given that treatment was three times per week. Hence more clinical research is needed on the effect of delayed treatment with EPO (e.g., onset at 2 days after birth).

Of note is that treatment with EPO alone yielded promising neurological outcomes in preterm infants treated for anemia at 27–30 weeks-of gestation [146] and after neonatal HIE at term [114,116,119]. For neonatal HIE at term, this treatment could be effective in resource poor situations where HT is unavailable [119]. The outcome of EPO treatment for preterm infants at 27 weeks-of-gestation or older may also be useful for neonatal HIE. This is because neonatal HIE can be associated with preceding brain injury (e.g., from infection or impaired placental functioning) during pregnancy.

For treatment with EPO alone, and for combined treatment with EPO and HT, the critical importance of animal experiments on the specific dose, timing, and frequency of EPO treatment prior to commencing clinical trials is evident from this review of the literature. The literature also reveals that the severity of the brain injury and gender can influence the effectiveness of therapy using either EPO alone or combined EPO and HT. Future studies require consideration of these critical contexts.

## Figures and Tables

**Table 1:** Animal studies on neonatal hypoxia-ischemia or neonatal stroke: Effect of EPO alone.

Study	Animal Model, Age and Species	Gender	Dose	Type of EPO	Timing of Administration	Route of Administration	Key Findings: *Short-Term Findings in Italics*
Matsushita et al. 2003 [71]	Rice-Vannucci, PN7 mice	Unknown	1 U/g or 5 U/g	Unknown	1 h before hypoxia	Intraperitoneal (i.p.)	*Decreased infarct volume or cerebral injury score at 24 h and 7 days post-injury*
Aydin et al. 2003 [72]	Rice-Vannucci, PN 7 rat	Unknown	20 U	r-Hu-EPO	Immediate post-treatment	Intracerebroventricular (ICV)	*EPO decreased brain infarct volume at 7 days post-injury*
Kumral et al. 2003 [73]	Rice-Vannucci, PN7 rat	Unknown	1000 U/kg	r-Hu-EPO	Immediate post-treatment	i.p.	*EPO decreased brain infarct volume at 3 days post-injury*
Kumral et al. 2004 [48]	Rice-Vannucci, PN7 rat	Unknown	1000 U/kg	r-Hu-EPO	Immediate post-treatment	i.p.	*Decreased overproduction of cerebral nitric oxide*
Kumral et al. 2005 [53]	Rice-Vannucci, PN7 rat	Unknown	1000 U/kg	r-Hu-EPO	Immediate post-treatment	i.p.	*Decreased lipid peroxidation;* *Increased activity of antioxidant enzyme*
Kumral et al. 2004 [60]	Rice-Vannucci, PN7 rat	Unknown	1000 U/kg	r-Hu-EPO	Immediate post-treatment	i.p.	20 weeks post-injury: Improved memory and cerebral volume
McClure et al. 2007 [82]	Rice-Vannucci, PN7 rat	Male	300 or 1000 U/kg	Unknown	Immediate post-treatment	i.p.	2–3 months post-injury: Improved memory, rapid auditory processing
Alexander et al. 2012 [83]	Rice-Vannucci, PN 7 rat	Male	1000 U/kg	Unknown	Immediate post-treatment, or delayed treatment at 1 h or 3 h	i.p.	Immediate, but not delayed, treatment with EPO has therapeutic benefit for auditory processing
Spandou et al. 2005 [84]	Rice-Vannucci, PN 7 rat	Unknown	2000 U/kg	Unknown	Immediate post-treatment	i.p.	6 weeks post-injury: Improved sensorimotor function; Reduced brain damage
Demers et al. 2005 [85]	Rice-Vannucci, PN 7 rat, 1.5 hypoxia	Unknown	2500 U/kg	rEPO	Repeated daily, 3 days post-injury	Subcutaneous (s.c.)	3 weeks post-injury: Prevented rotation, reduced sensory neglect. No effect on overall brain injury score.
van der Kooij et al. 2009 [86]	Rice-Vannucci, PN 7 rat, 1.5 hypoxia	Unknown	1000 U/kg	r-Hu-EPO, epoetin-alfa	Repeated daily, 0 h, 24 h, 48 h post-injury	i.p.	No effect on cerebral white or grey matter damage
Fan et al. 2011 [87]	Rice-Vannucci, PN 9 mice, 45 min hypoxia	Male and female	5000 U/kg	Unknown	Repeated daily, 0 h, 24 h, 48 h post-injury	i.p.	*At 72 h, Increased progenitor cells in subventricular zone and dentate gyrus in females but not males*
Wen et al. 2006 [88]	Neonatal stroke, PN7 rat	Male and female	1000 U/kg	Unknown	Repeated daily, 15 min, 24 h, 48 h post-injury	i.p.	Long-term neuroprotection-more beneficial in females
Sola et al. 2005 [89]	Neonatal stroke, PN7 rat	Unknown	100 or 1000, 5000 U/kg	r-Hu-EPO	Repeated, after 15 min and on days 1 and 2 post-stroke	i.p.	Reduced cerebral infarct volume at 3 days post-stroke
Chang et al. 2005 [90]	Neonatal stroke, PN10 rat	Unknown	5000 U/kg	r-Hu-EPO	Immediately after hypoxia	i.p.	Short-term improvements in sensorimotor outcomes
Gonzalez et al. 2009 [91]	Neonatal stroke, PN10 rat	Unknown	1000 U/kg	r-Hu-EPO	Repeated: 0 h, 24 h, 7 days post-injury	i.p.	No long-term difference in memory at 3-months compared to shams
Gonzalez et al. 2013 [92]	Neonatal stroke, PN7 rat	Unknown	1000 U/kg	r-Hu-EPO	Repeated: 0 h, 24 h, 7 days post-injury	i.p.	Increased neurogenesis and oligodendrogenesis after EPO
Kellert et al. 2007 [93]	Rice-Vannucci, PN 7 rat	Unknown	5000 or 30,000 U/kg	r-Hu-EPO, epoetin-alfa	Repeated: 1, 3 or 7 daily injections, started immediately after hypoxia	s.c.	Maximal benefit against brain injury with 3 doses of 5000 U/kg or 1 dose of 30,000 U/kg
Iwai et al. 2007 [94]	Rice-Vannucci, PN 7 rat	Unknown	1000 U/kg	r-Hu-EPO	Repeated: 20 min, 2, 4 & 6 days post-injury	i.p.	*EPO decreased cerebral infarct volume 1 week later*, and improved motor outcomes 4 weeks later
Sun et al. 2005 [95]	Rice-Vannucci, PN 7 rat	Unknown	5000 U/kg	r-Hu-EPO	Repeated: 24 h, 48 h, 72 h post-injury	i.p.	EPO neuroprotective and decreased the inflammatory response
Iwai et al. 2010 [96]	Rice-Vannucci, PN 7 rat	Unknown	1000 U/kg	r-Hu-EPO	Repeated: 2, and for 4 days until 13 days post-injury	i.p.	EPO did not prevent brain volume loss, but improved oligodendro-genesis and sensorimotor function at day 14 post-injury
Chen et al. 2012 [97]	Rice-Vannucci, PN 10 rat	Unknown	300 U/kg	Nano-EPO	Repeated: 1 h, 48 h, 48 h post-injury	i.p.	*Similar outcomes to 5000 U/kg: EPO neuroprotective at 72 h post-injury*, and improved functional deficit at 21 days post-injury

**Table 2:** Animal studies on neonatal hypoxia-ischemia: Effect of EPO in combination with moderate hypothermia.

Study	Animal Model, Age and Species	Gender	Dose	Type of EPO	Timing of Administration	Route of Administration	Key Findings
Fan et al. 2013 [105]	Rice-Vannucci, PN7 rat	Males and females	5000 U/kg	EPREX	Immediately after 3 h of hypothermia (4 °C decrease)	Intraperitoneal (i.p.)	Combined treatment had only a borderline (*p* = 0.07) neuroprotective effect at 6 weeks-of-age
Fang et al. 2013 [106]	Rice-Vannucci, PN 7 rat	Unknown	1000 U/kg	r-Hu-EPO	EPO post-treatment at 0 h, 24 h and 7 days; hypothermia at 1–9 h post-hypoxia	i.p.	No adverse effects of combined treatment at 2- and 6-weeks post-injury
Traudt et al. 2013 [107]	Non-human primate model of perinatal asphyxia	Unknown	1000, 2500 or 3500 U/kg	r-Hu-EPO	EPO post-treatment at 0.5 h, 24 h, 48 h and 7 days; hypothermia at 3–75 h post-hypoxia	Intravenous (i.v.); Either at 3500 U/kg for the first dose and then 2500 U/kg thereafter or at 1000 U/kg for all four doses	Hypothermia combined with 4 doses of EPO significantly decreased the risk of cerebral palsy at 9 months-of-age
McAdams et al. 2017 [108]	Non-human primate model of perinatal asphyxia	Unknown	1000, 2500 or 3500 U/kg	r-Hu-EPO	EPO post-treatment at 0.5 h, 24 h, 48 h and 7 days; hypothermia at 3–75 h post-hypoxia	i.v.	Hypothermia combined with 4 doses of EPO significantly decreased neuropathology at 9 months-of-age

**Table 3:** Clinical studies on neonatal hypoxic-ischemic encephalopathy: Effect of EPO alone.

Study	Number of Term Newborn Infants	Gender	Dose	Type of EPO	Timing of Administration	Route of Administration	Key Findings
Zhu et al. 2009 [114]	167, born between August 2003 and January 2007, with either moderate or severe hypoxic-ischemic encephalopathy (HIE)	Males and females	300 or 500 U/kg (*n* = 83) or con-ventional (*n* = 84)	r-Hu-EPO	1–48 h after birth for 1st dose; then every other day for 2 weeks	Subcutaneously (s.c.) for 1st dose; Intravenous (i.v.) thereafter	When 18-months-old, improved long-term outcomes after EPO treatment in the infants with moderate HIE, but not in those with severe HIE
Elmahdy et al. 2010 [116]	45, 3 groups: normal (*n* = 15), HIE with conventional treatment * (*n* = 15), or HIE with EPO treatment (*n* = 15)	Males and females	2500 U/kg	r-Hu-EPO	4–6 h after birth for 1st dose; then daily for 4 days	s.c.	When 2-weeks-old for HIE infants, EPO decreased nitric oxide concentration and breakthrough seizures compared to conventional treatment. When 6-month-old for HIE infants, EPO decreased neurologic and developmental abnormalities.
Avasiloaiei et al. 2013 [117]	67, 3 HIE groups treated with EPO & supportive care ** (*n* = 22), or phenobarbital (*n* = 22) or supportive care alone (*n* = 23)	Unknown	1000 IU/kg	EPO	EPO post-treatment during the 1st 3 days	s.c.	When 18-months-old, neurodevelop-mental delay was lower in both the EPO and phenobarbital treatment groups, although the differences were not statistically analyzed
El Shimi et al. 2014 [118]	45, HIE/EPO (*n* = 15), HIE/hypothermia (*n* = 15), normal (*n* = 15)	Males and females	1500 U/kg	r-Hu-EPO	Single dose on postnatal day 1	s.c.	When 3-months-old, no significant differences in neuromuscular function nor brain MRI score
Malla et al. 2017 [119]	100, HIE/EPO (*n* = 50), HIE/placebo (saline, *n* = 50)	Males and females	500 U/kg	r-Hu-EPO	1st dose within 6 h of birth; then every other day for a total of 5 doses	i.v.	When 19-months-old, the EPO-treated group had a lower risk of cerebral palsy. EPO also decreased death

* Conventional treatment in most neonatal centers includes respiratory support, fluid infusion, anti-convulsants, reducing intracranial pressure, ionotropic support to maintain blood pressure and the correction of hypoglycemia, acidosis and electrolyte imbalance [115]. ** Supportive care was defined as ‘oxygen, volume expanders, ionotropes, diurectics, and antibiotics’.

**Table 4:** Clinical studies on neonatal hypoxic-ischemic encephalopathy: Effect of EPO combined with moderate hypothermia.

Study	Number of Term Newborn Infants	Gender	Dose	Type of EPO	Timing of Administration	Route of Administration	Key Findings
Baserga et al. 2015 [122]	30, with hypoxic-ischemic encephalopathy (HIE), 3 groups, placebo (*n* = 10), EPO (low dose, *n* = 10), EPO (high dose, *n* = 10)	Males and females	2 or 10 U/kg	darbe-poietin	EPO within 12 h of birth, and a 2nd dose 7 days later; Hypothermia started within 11 h of birth and given for 72 h	Intravenous (i.v.)	HT combined with EPO was safe. Weekly administration of darbepoietin was sufficient
Valera et al. 2015 [123]	15, HIE and treated with EPO and moderate hypothermia	Males and females	400 U/kg	r-Hu-EPO	Every 48 h for 2 weeks, commencing within 3 h of birth, along with hypothermia for 72 h	i.v.	When 18-months-old, 80% survival with no neurodevelopmental disability. Unfortunately, no control group
Rogers et al. 2014 [124]	24, HIE and treated with EPO and moderate hypothermia; 250 U/kg EPO (*n* = 3), 500 (*n* = 6), 1000 (*n* = 7), 2500 (*n* = 8)	Males and females	250 to 2500 U/kg	r-Hu-EPO	EPO at 24 h after birth, and then every 48 h; Hypothermia started within 6 h of birth and given for 72 h	i.v.	When 8-34-months-old, neurodevelopmental delay was lower compared to treatment with hypothermia alone. However, study statistically underpowered to detect a statistical difference
Mulkey et al. 2017 [125]	44, HIE; In addition to treatment with moderate hypothermia, treated with EPO (*n* = 20) or saline (*n* = 24)	Males and females	1000 U/kg	r-Hu-EPO	EPO on postnatal day 1 (at <24 h), 2&3 (*n* = 11), plus day 5 (*n* = 8), plus day 7 (*n* = 1); Hypothermia started within 6 h of birth and given for 72 h	i.v.	Statistically significant lower volume of acute brain injury in the EPO-hypothermia-treated group compared with the saline-hypothermia group
Wu et al. 2016 [126]	50, HIE; In addition to treatment with moderate hypothermia, treated with EPO (*n* = 24) or saline (*n* = 26)	Males and females	1000 U/kg	r-Hu-EPO	EPO on postnatal day 1 (at <24 h), 2, 3, 5 and 7; Hypothermia started within 6 h of birth and given for 72 h	i.v.	Significantly less brain injury at 5 days-of-age, and better 12-month motor outcomes, in the EPO-hypothermia-treated group compared with the saline-hypothermia group
Juul et al. 2018 [130]	Recruiting 500, HIE; In addition to treatment with moderate hypothermia, treated with EPO or saline	Males and females	1000 U/kg	r-Hu-EPO	EPO on postnatal day 1 (at <24 h), 2, 3, 5 and 7; Hypothermia started within 6 h of birth and given for 72 h	i.v.	Assessment up to 24 months-of-age. Hypothesize that EPO in combination with hypothermia reduces mortality and neurodevelopmental disability
Patkai et al. 2014 [132]	Recruiting 120, HIE; In addition to treatment with moderate hypothermia, treated with EPO or saline	Males and females	1000–1500 U/kg	beta r-Hu-EPO	EPO on postnatal day 1 (at <12 h), 2 and 3 (each 24 h after previous dose); Hypothermia started within 6 h of birth and given for 72 h	i.v.	Assessment up to 24 months-of-age. Hypothesize that EPO in combination with hypothermia increases survival and reduces neurological sequelae
Wang 2017 [134]	68, with HIE, 2 groups, moderate hypothermia, EPO & Vitamin C (*n* = 34), EPO & Vitamin C (*n* = 34)	Males and females	500 U/kg	r-Hu-EPO	EPO given 3 times per week (start time unknown); Vitamin C given once per day; Hypothermia given for 72 h	i.v. for EPO and Vitamin C (250 mg/kg)	Mild hypothermia, EPO & Vitamin C combined more effective. This was achieved through decreased apoptosis and oxygen free radicals, and increased antioxidant capacity
Nonomura et al. 2019 [135]	9, with severe HIE, moderate hypothermia, EPO & magnesium sulfate (Mg)	Males and females	300 U/kg	Epoietin alfa	EPO & Mg given within 6 h of birth, then every other day for 2 weeks. Hypothermia started within 6 h of birth, for 72 h	i.v. for EPO and Mg (250 mg/kg)	No deaths and all 9 neonates did not have any serious adverse effects
